# The Role of SDF-1-CXCR4/CXCR7 Axis in the Therapeutic Effects of Hypoxia-Preconditioned Mesenchymal Stem Cells for Renal Ischemia/Reperfusion Injury

**DOI:** 10.1371/journal.pone.0034608

**Published:** 2012-04-12

**Authors:** Hongbao Liu, Shuibing Liu, Yang Li, Xiaohong Wang, Wujun Xue, Guanqun Ge, Xiaohui Luo

**Affiliations:** 1 Department of Nephrology, Xijing Hospital, Fourth Military Medical University, Xi'an, China; 2 Department of Pharmacology, School of Pharmacy, Fourth Military Medical University, Xi'an, China; 3 Department of Renal Transplant, The First Affiliated Hospital, Xi'an Jiaotong University, Xi'an, China; National Cancer Institute, United States of America

## Abstract

*In vitro* hypoxic preconditioning (HP) of mesenchymal stem cells (MSCs) could ameliorate their viability and tissue repair capabilities after transplantation into the injured tissue through yet undefined mechanisms. There is also experimental evidence that HP enhances the expression of both stromal-derived factor-1 (SDF-1) receptors, CXCR4 and CXCR7, which are involved in migration and survival of MSCs *in vitro*, but little is known about their role in the *in vivo* therapeutic effectiveness of MSCs in renal ischemia/reperfusion (I/R) injury. Here, we evaluated the role of SDF-1-CXCR4/CXCR7 pathway in regulating chemotaxis, viability and paracrine actions of HP-MSCs *in vitro* and *in vivo*. Compared with normoxic preconditioning (NP), HP not only improved MSC chemotaxis and viability but also stimulated secretion of proangiogenic and mitogenic factors. Importantly, both CXCR4 and CXCR7 were required for the production of paracrine factors by HP-MSCs though the former was only responsible for chemotaxis while the latter was for viability. SDF-1α expression was upregulated in postischemic kidneys. After 24 h systemical administration following I/R, HP-MSCs but not NP-MSCs were selectively recruited to ischemic kidneys and this improved recruitment was abolished by neutralization of CXCR4, but not CXCR7. Furthermore, the increased recruitment of HP-MSCs was associated with enhanced functional recovery, accelerated mitogenic response, and reduced apoptotic cell death. In addition, neutralization of either CXCR4 or CXCR7 impaired the improved therapeutic potential of HP-MSCs. These results advance our knowledge about SDF-1-CXCR4/CXCR7 axis as an attractive target pathway for improving the beneficial effects of MSC-based therapies for renal I/R.

## Introduction

Renal ischemia/reperfusion (I/R) injury is the most common cause for acute kidney injury (AKI) which affects both native and transplanted kidneys and is associated with high morbidity and mortality [1], [2]. A large number of studies have focused on the endogenous and exogenous mechanisms of kidney repair after schemic/hypoxic injury [3]–[6]. Interestingly, a recent clinical study suggested that nocturnal hypoxia was associated with accelerated loss of kidney function in patients with obstructive sleep apnea syndrome [7]. In the last few years, several studies have shown that mesenchymal stromal cells (MSCs) can prevent or attenuate ischemic tissue injury, possibly by paracrine/autocrine mechanisms or trans-differentiation into local cell types [5], [6], [8]–[13]. When administered systemically, however, only a small proportion of the infused MSCs homing to the ischemic tissue, whereas the majority of cells were found entrapped in other organs including lungs [10], [14], [15]. Furthermore, due to the local hypoxia, oxidative stress and inflammation in the targeted ischemic tissue, the retention of transplanted MSCs is poor and the low cell survival reduces the therapeutic effects [16]. Thus, it is crucial to find techniques which can enhance the chemotaxis and retention of the implanted MSCs to maximize the effectiveness of MSC-based therapy.

The ischemic tissue produces numerous cytokines, chemokines, secreted proteins and growth factors that may influence organ-specific and stem cell-mediated repair [17]–[22]. Several studies also provided evidences for a critical role of hypoxia-inducible factors in renal epithelial differentiation and repair [23]–[26]. A number of studies have proven that chemokine stromal cell-derived factor-1 (SDF-1, also known as CXCL12) is critical for the process involving stem/progenitor cell chemotaxis and organ-specific homing in ischemic tissue through interaction with its cognate receptor CXC chemokine receptor 4 (CXCR4) on the surface of stem/progenitor cells [27]–[31]. Although CXCR4 is highly expressed in MSCs within the bone marrow, its expression is markedly reduced during ex vivo expansion of MSCs [32], [33]. This could decrease the ability of implanted MSCs to respond to homing signals emanated from the ischemic tissue [34]. Several reports have showed that short-term exposure of MSCs to hypoxia could upregulate CXCR4 expression [35]–[38]. We have previously reported that CXCR7, a novel receptor for SDF-1, is also upregulated by sub-lethal hypoxic preconditioning (HP) of cultured MSCs [39]. However, the effect of both SDF-1 receptors on paracrine actions of MSCs still remains unknown; especially, the role of the SDF-1-CXCR4/CXCR7 axis in the therapeutic effects of MSCs for renal I/R has not been evaluated *in vivo*.

In this study, we demonstrate for the first time that HP markedly augments chemotaxis, viability and paracrine actions of MSCs *in vitro* and thus enhances the benefit of MSC-based therapy for renal I/R through interaction of SDF-1 with CXCR4 and CXCR7.

## Results

### HP upregulates expression of SDF-1α and its receptors, CXCR4 and CXCR7, in MSCs

**Figure 1:**
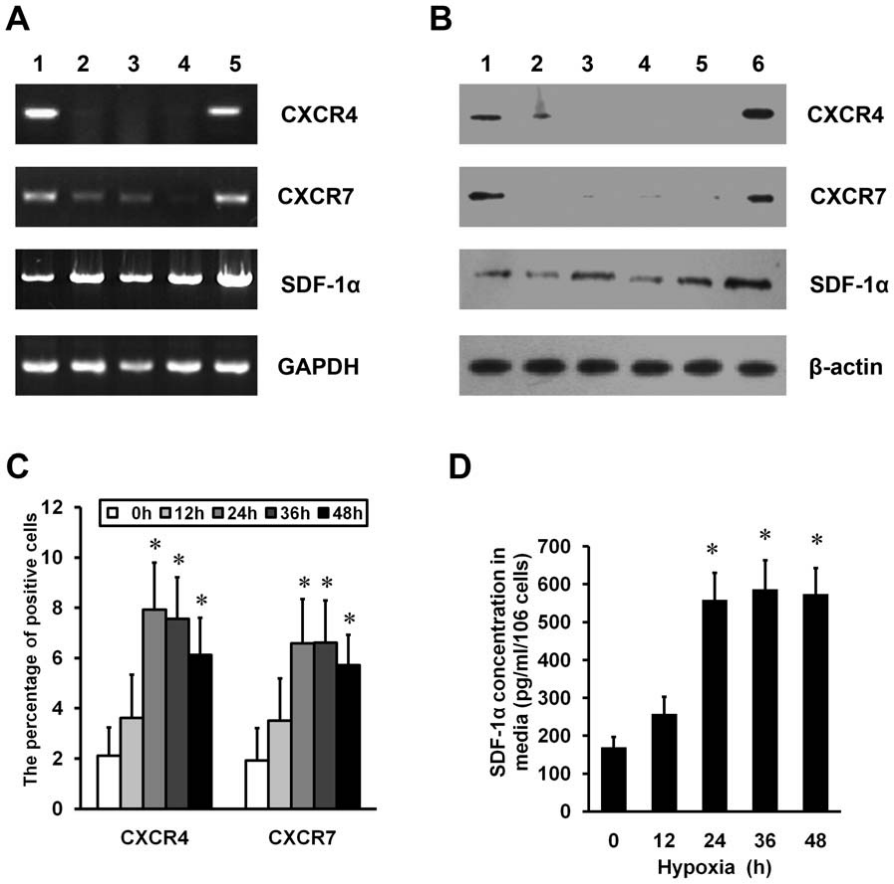
Effects of HP on the expression of SDF-1α, CXCR4, CXCR7 in MSCs. (A) Semiquantitative RT-PCR was used for the analysis of SDF-1α, CXCR4 and CXCR7 mRNA levels in MSCs. GAPDH was used as a control. Lane 1 indicates bone marrow mononuclear cells (BMMCs); lanes 2 to 4, MSC cultures at passage 1 to 3; and lane 5, MSCs at passage 3 and exposed to hypoxia (3% O_2_) for 24 h. (B) Western blot analysis was performed to detect CXCR4, CXCR7 and SDF-1α protein expression. β-actin was used as a control. Lanes 1 indicates BMMCs; lanes 2 to 5, MSC cultures at passage 1 to 4; and lane 6, MSCs at the third passage to hypoxia for 24 h. (C) FCM was used to detect extracellular expression of CXCR4 or CXCR7 in MSCs exposed to the indicated periods of hypoxia. *P<0.05, vs 0 h. (D) ELISA analysis was performed to determine production of SDF-1α from MSCs exposed to the indicated periods of hypoxia. *P<0.05, vs 0 h.

Preliminary experiments showed that the viability and growth of MSCs were not adversely affected by 48 h of hypoxia (3% O_2_) (data not shown). The mRNA level and protein expression of CXCR4 and CXCR7 are high in bone marrow mononuclear cells, but low or undetectable in MSCs at passage 1 to 3 (Fig. 1A and B). The exposure of MSCs at passage 3 to hypoxia for 24 h upregulated the expression of SDF-1α and its receptors (Fig. 1A and B). To examine cell surface expression of CXCR4 and CXCR7, flow cytometry (FCM) was performed and revealed that number of either CXCR4- or CXCR7-positive cells was significantly higher in MSCs exposed to hypoxia for 24 h, 36 h and 48 h than that for 0 h, respectively (Fig. 1C). Furthermore, enzyme-linked immunosorbent assay (ELISA) analysis showed HP caused a time-dependent increase of SDF-1α protein level, reaching maximal at 24 h to 48 h after HP (Fig. 1D).

### SDF-1-CXCR4 axis is required for MSC chemotaxis

**Figure 2:**
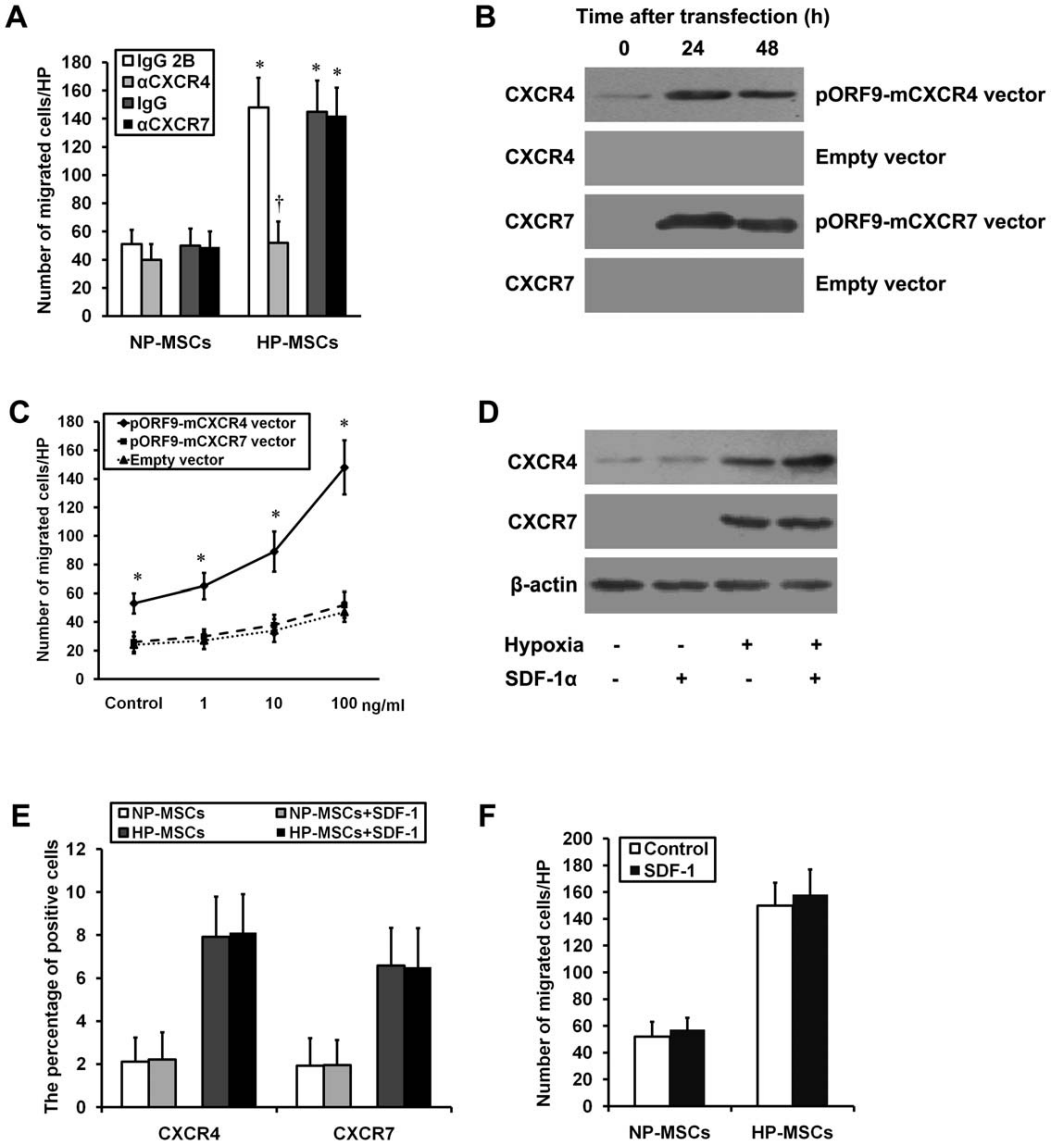
Effects of SDF-1-CXCR4/CXCR7 pathway on MSC chemotaxis in vitro. (A) The chemotaxis in response to SDF-1α (10 ng/ml for 12 h) was performed in the NP-MSCs and HP-MSCs treated with a neutralizing anti-CXCR4 antibody, an anti-CXCR7 antibody, and the respective isotype-matched control antibodies. *P<0.05, vs NP-MSCs; ^†^ *P*<0.05, vs the respective isotype-matched control antibodies. (B) NP-MSCs were transiently overexpressed with CXCR4 using pORF9-mCXCR4 vector or with CXCR7 using pORF9-mCXCR7 vector (*n* = 6). A negative control empty (pORF9-MCS) vector was used. (C) The transfected cells were subjected to chemotaxis in response to the indicated concentrations of SDF-1α for 12 h. *P<0.05, vs the empty vector. (D and E) Western blot analysis (D) and FCM (E) were performed to determine the intracellular and extracellular expression of both CXCR4 and CXCR7 in the cells treated with or without SDF-1α (50 ng/ml for 60 min). (F) The chemotaxis in response to SDF-1α was performed in the cells treated with or without SDF-1α.

In accord with our previous study [39], the present study demonstrated that HP significantly increased MSC chemotaxis in response to SDF-1α, and this increased chemotaxis was blocked obviously by an anti-CXCR4 antibody, but not by an anti-CXCR7 antibody (Fig. 2A). To further support this possibility, NP-MSCs where both CXCR4 and CXCR7 expression was undetectable were transfected with sense expression vectors of pORF9-mCXCR4 or pORF9-mCXCR7, or empty vector pORF9, respectively. Numerous clones showing increased CXCR4 or CXCR7 expression were screened by the level of expression of either CXCR4 or CXCR7 and confirmed by western blots after 24 h and 48 h of transfection (Fig. 2B). Following transfection, cells were subjected to 24 h of normoxia followed by 6 h of 1–100 ng/ml SDF-1α treatment. As expected, there was a dose-dependent increase in the chemotaxis in response to SDF-1α in CXCR4-transfected cells, but not in CXCR7-transfected and empty vector-transfected cells (Fig. 2C). Furthermore, as shown in Fig. 2D through [Fig pone-0034608-g003] 4F, SDF-1α stimulation (50 ng/ml) had no effect on the chemotaxis in response to SDF-1α and the expression of CXCR4 and CXCR7 in both HP-MSCs and NP-MSCs.

**Figure 3 pone-0034608-g003:**
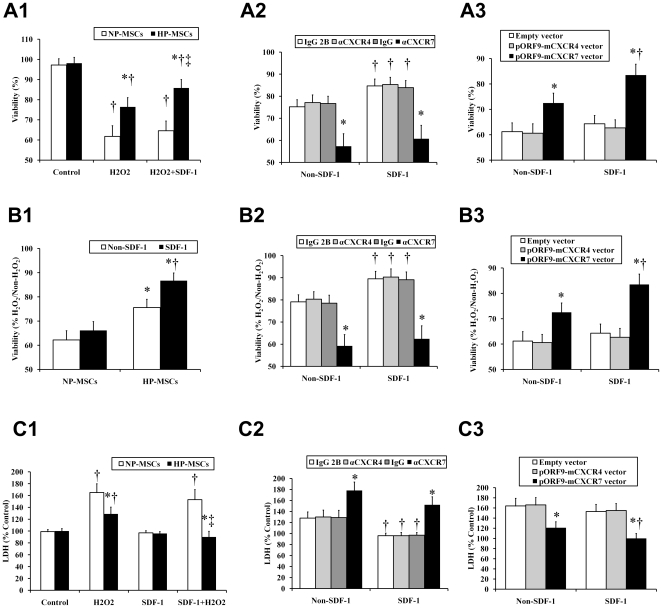
Effects of SDF-1-CXCR4/CXCR7 pathway on H_2_O_2_-induced cytotoxicity in MSCs. The standard cytotoxicity tests, including propidium iodide (PI)-based cell viability (A1–A3), MTT assay for mitochondrial viability (B1–B3), LDH assay for membrane damage (C1–C3), were performed. (A1, B1, and C1) MSCs were incubated in H_2_O_2_-conditioned media (250 µM) added with or without SDF-1α (50 ng/ml) for 6 h. The cells incubated in absence of both H_2_O_2_ and SDF-1α were used as control. A1 and C1: *P<0.05, vs NP-MSCs; ^†^ *P*<0.05, vs Control; ^‡^ *P*<0.05, vs H_2_O_2_. B1: *P<0.05, vs NP-MSCs; ^†^ *P*<0.05, vs Non-SDF-1. (A2, B2, and C2) Prior to H_2_O_2_ treatment, HP-MSCs were treated with a neutralizing anti-CXCR4 antibody, an anti-CXCR7 antibody, and the respective isotype-matched control antibodies, respectively. *P<0.05, vs the respective isotype-matched control antibodies; ^†^ *P*<0.05, vs non-SDF-1. (A3, B3, and C3) Prior to H_2_O_2_ treatment, NP-MSCs were transiently overexpressed with CXCR4 using pORF9-mCXCR4 vector or with CXCR7 using pORF9-mCXCR7 vector. *P<0.05, vs empty vector; ^†^ *P*<0.05, vs Non-SDF-1.

**Figure 4:**
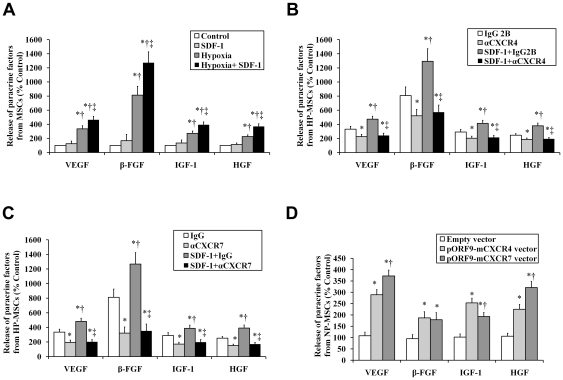
Effects of SDF-1-CXCR4/CXCR7 pathway on MSC paracrine actions. ELISA was performed to determine production of VEGF, β-FGF, IGF-1 and HGF from MSCs stimulated by hypoxia (3% O_2_) or/and SDF-1α (50 ng/ml). The cells stimulated by neither hypoxia nor SDF-1α were used as control. (A) MSCs were stimulated with hypoxia or/and SDF-1α. *P<0.05, vs control; ^†^ *P*<0.05, vs SDF-1; ^‡^ *P*<0.05, vs hypoxia. (B and C) The HP-MSCs stimulated with or whthout SDF-1α were treated with an anti-CXCR4 antibody (B), an anti-CXCR7 antibody (C), and the respective isotype-matched control antibodies. *P<0.05, vs IgG2B (B) or IgG (C); ^†^ *P*<0.05, vs αCXCR4 (B) or αCXCR7 (C); ^‡^ *P*<0.05, vs SDF-1+IgG2B (B) or SDF-1+IgG (C). (D) NP-MSCs were transiently overexpressed with CXCR4 using pORF9-mCXCR4 vector or with CXCR7 using pORF9-mCXCR7 vector. *P<0.05, vs empty vector; ^†^ *P*<0.05, vs pORF9-mCXCR4 vector.

### SDF-1-CXCR7 axis is required for MSC viability

Since H_2_O_2_ has previously been shown to be a critical mediator of hypoxia/reoxygenation- or ischemia/reperfusion-induced cell death [40], we investigated the effect of HP on H_2_O_2_-induced cytotoxicity of MSCs. To this goal, standard cytotoxicity tests, including MTT assay for mitochondrial viability, propidium iodide (PI)-based cell viability, and LDH assay for membrane damage, were performed. The results of cell viability assays by an automated NucleoCounter (Fig. 3A1) revealed no apparent cytotoxicity in HP-MSCs compared with that in NP-MSCs under normal culture conditions. H_2_O_2_ treatment increased the cytotoxicity in both NP-MSCs and HP-MSCs, however, the increase was more dramatic in NP-MSCs than in HP-MSCs (Fig. 3A1, B1 and C1). Pretreatment of HP-MSCs with an anti-CXCR7 antibody but not with an anti-CXCR4 antibody completely increased the H_2_O_2_-induced cytotoxicity in comparison with cells treated with the respective isotype matched control antibodies (Fig. 3A2, B2 and C2). Contrarily, the H_2_O_2_-induced cytotoxicity was significantly decreased in CXCR7-transfected NP-MSCs compared with the CXCR4-transfected and empty vector-transfected cells (Fig. 3A3, B3 and C3).

In addition, the role of SDF-1α on H_2_O_2_-induced cytotoxicity of MSCs was also evaluated. Addition of SDF-1α (50 ng/ml) to the culture had no effect on cell viability and LDH release of NP-MSCs under H_2_O_2_ culture conditions (Fig. 3A1, B1 and C1). However, treatment with SDF-1α markedly protected HP-MSCs against H_2_O_2_ when compared with non SDF-1α treatment (Fig. 3A1, B1 and C1). Importantly, this SDF-1α-induced protection for HP-MSCs was obviously blocked by an anti-CXCR7 antibody, but not by an anti-CXCR4 antibody (Fig. 3A2, B2 and C2). Furthermore, transfection of CXCR7 markedly protected NP-MSCs against H_2_O_2_, as indicated by SDF-1α-induced increase in nuclear/mitochondrial viability and decrease in LDH release when compared with transfection of CXCR4- or empty vector (Fig. 3A3, B3 and C3).

### Both SDF-1-CXCR4 axis and SDF-1-CXCR7 axis are required for MSC paracrine actions

We first determined the effects of HP on MSCs-secreted proangiogenic and mitogenic factors. We found that 24 h of hypoxia significantly increased MSC-secreted vascular endothelial growth factor (VEGF), β-fibroblast growth factor (β-FGF), insulin-like growth factor 1 (IGF-1), and hepatocyte growth factor(HGF) compared with normoxia (Fig. 4A). Furthermore, the production of these factors in the presence of SDF-1α was enhanced markedly in HP-MSCs but only slightly in NP-MSCs (Fig. 4A).

Next, we wanted to determine the role of CXCR4 and CXCR7 in MSC paracrine actions. As shown in Fig. 4B and 4C, HP-induced secretion of growth factors was completely abolished by blocking either CXCR4 receptor with an anti-CXCR4 antibody or CXCR7 receptor with an anti-CXCR7 antibody. Contrarily, the secretion of these factors was significantly increased in CXCR4- or CXCR7-transfected NP-MSCs compared with the empty vector-transfected cells (Fig. 4D).

### SDF-1α expression is upregulated in postischemic kidneys

**Figure 5:**
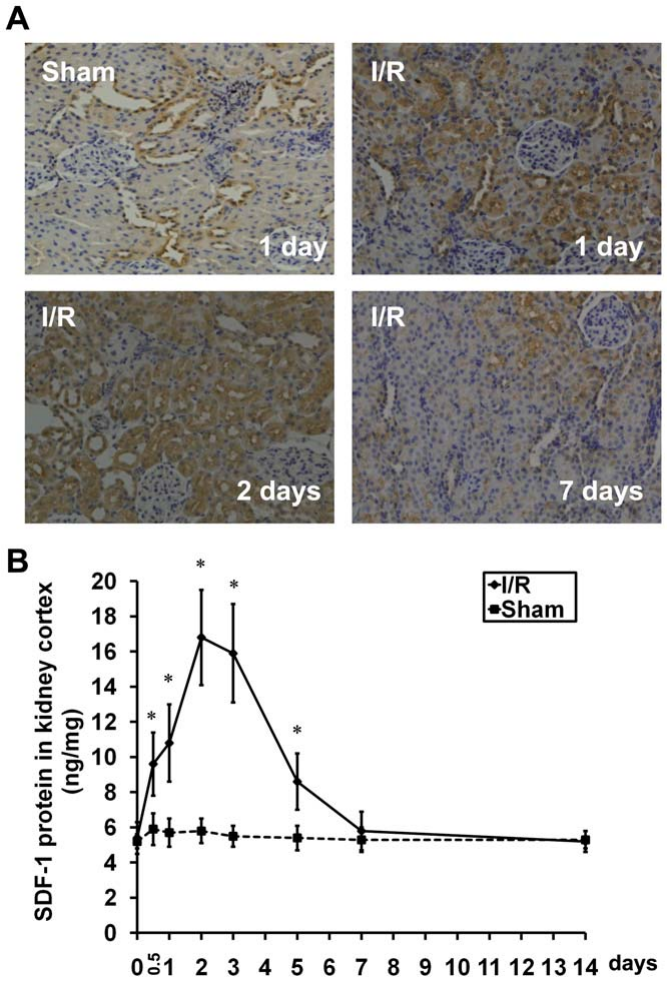
SDF-1α is upregulated in the kidney of I/R-AKI mice. (A) Representative micrographs of immunohistochemistry for SDF-1α in the kidneys from mice affected by IR-AKI days 1, 2 and 7 after I/R. The kidney sections from mice 24 h after sham surgery were used as control (upper left panel). Original magnification ×200. (B) The kidney cortex lysates from mice affected by sham surgery or I/R-AKI were analyzed by ELISA to determine SDF-1α protein expression at the indicated periods of post-surgery time. *P<0.05, vs Sham.

We assessed SDF-1α expression in the kidney obtained from mice treated with either sham or I/R surgery. Immunohistochemistry staining showed that SDF-1α was extensively expressed in the cytoplasm of renal tubules cells in I/R-AKI mice, but only sporadically expressed in sham-operated kidneys (Fig. 5A). SDF-1α appeared to be increased within the first 24 h of I/R, peaked at 48 h and rapidly downregulated in the subsequent days till day 7 after injury to the level comparable to that observed in sham-operated kidneys (Fig. 5A). In addition, incubation with secondary antibody alone did not result in any staining (data not shown). Moreover, similar results of SDF-1α protein levels in the kidney from I/R-AKI mice were also observed by ELISA (Fig. 5B).

### HP-MSCs migrate toward hypoxia/reoxygenation-damaged renal tubule epithelial cells in vitro

**Figure 6:**
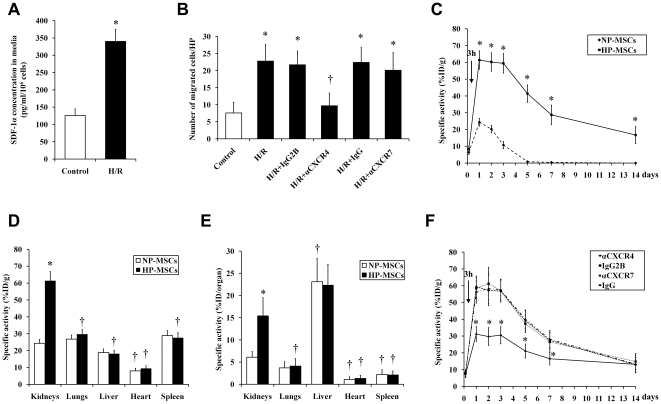
The role of SDF-1-CXCR4/CXCR7 pathway on the homing of HP-MSCs toward ischemic kidneys. (A) ELISA analysis was performed to determine production of SDF-1α from primary TECs exposed to hypoxia/reoxygenation in vitro. The cells without hypoxia/reoxygenation stimulation were used as control. *P<0.05, vs control. (B) The chemotaxis in response to hypoxia/reoxygenation-damaged primary TECs was performed in HP-MSCs treated with a neutralizing anti-CXCR4 antibody, an anti-CXCR7 antibody, and the respective isotype-matched control antibodies, respectively. *P<0.05, vs control; ^†^ *P*<0.05, vs the respective isotype-matched control antibodies. (C) The kidney uptake of ^111^Indium-labeled NP-MSCs and HP-MSCs was measured from 3 hours to 14 days after systemic administration into I/R-AKI mice. The specific radioactivity of each organ was expressed as the percentage of injected dose per gram tissue (%ID/g) after being adjusted by the half-life of ^111^Indium. *P<0.05, vs the respective isotype-matched control antibodies. (D and E) The uptake of ^111^Indium-labeled NP-MSCs and HP-MSCs in different organs from I/R-AKI mice was measured 24 h after infusion. *P<0.05 vs NP-MSCs; ^†^ *P*<0.05 vs Kidney. (F) The kidney uptake of ^111^Indium-labeled NP-MSCs and HP-MSCs was measured from 3 hours to 14 days after infusing cells pretreated with a neutralizing anti-CXCR4 antibody, an anti-CXCR7 antibody and the respective isotype-matched control antibodies, respectively. *P<0.05, vs the respective isotype-matched control antibodies.

Primary renal tubule epithelial cells (TECs) from 1–2-week old C56BL/6 mice were exposed to hypoxia/reoxygenation. This procedure led to a 270% increase of SDF-1α protein level compared to that of baseline as determined by ELISA (Fig. 6A). HP-MSCs expressing CXCR4 and CXCR7 in the upper chamber of transwell system showed enhanced chemotaxis toward the hypoxia/reoxygenation-damaged monolayer of tubular cells in the lower chamber when compared to that observed with undamaged cells (Fig. 6B). Importantly, blocking of CXCR4 but not CXCR7 significantly decreased this chemotaxis toward damaged TECs (Fig. 6B).

### HP increases the in vivo homing of transplanted MSCs to ischemic kidneys

To validate the homing of MSCs to the target tissue, the cells were radioactively labeled with ^111^Indium-oxine and then systemically administered via tail vein into I/R-AKI mice. To facilitate comparisons, the specific radioactivity of each organ was adjusted by the half-life of ^111^Indium-oxine (2.8 days) and calculated as the percentage of injected dose per gram tissue (%ID/g) and percentage injected dose per organ (%ID/organ). No significant difference in radioactivity of kidneys was observed between HP-MSC-transplantated (8.4±1.7%ID/g) and NP-MSC-transplantated mice (6.4±1.4%ID/g) 3 h after infusion (Fig. 6C). Meanwhile, the uptake of radioactively labeled MSCs was restricted primarily to the lungs [(151±11)%ID/g and (20.9±1.7)%ID/organ]. 24 h after MSC infusion, radioactively labeled MSCs were lost from the lungs [(29±5)%ID/g and (4.1±0.8)%ID/organ] and redistributed to organs including kidneys, spleen and liver (Fig. 6D and E). In the meantime, the amount of HP-MSCs in the ischemic kidneys was 2.5 times greater than that of NP-MSCs. In contrast, MSC accumulation in other organs did not differ between HP-MSC-transplantated and NP-MSC-transplantated mice. On day 3 after infusion, kidney uptake of radioactively labeled HP-MSCs with decay correction was not significantly different from that on day 1 after infusion; however, kidney uptake of radioactively labeled NP-MSCs with decay correction dropped to 42.8±9.6% of baseline (Fig. 6C). The evidence that the kidney uptake of radioactively labeled MSCs in HP-MSC-transplantated animals and the lack of uptake in the kidneys in NP-MSC-transplantated animals at 5 to 14 days after infusion suggests that this difference is likely due to NP-MSC loss in injured kidneys.

### Neutralization of CXCR4, but not of CXCR7, impairs the increased homing capability of HP-MSCs

To further investigate the role of SDF-1-CXCR4 and/or CXCR7 interaction in the *in vivo* homing of HP-MSCs to ischemic kidneys, the ^111^Indium-labeled cells before transplantation were pretreated with a neutralizing anti-CXCR4 antibody, an anti-CXCR7 antibody, or their isotype-matched control antibodies, respectively. At 3 hours to 14 days after cell infusion, the kidney uptake of radioactively labeled HP-MSCs pretreated with an anti-CXCR7 antibody and control cells did not vary significantly (Fig. 6F). However, an obvious decrease in kidney uptake of radioactively labeled HP-MSCs pretreated with an anti-CXCR4 antibody was observed relative to that of cells pretreated with its isotype-matched control IgG2B antibody at 1 to 7 days after transplantation (Fig. 6F).

### HP improves the therapeutic potential of MSCs for treatment of I/R-AKI

**Figure 7:**
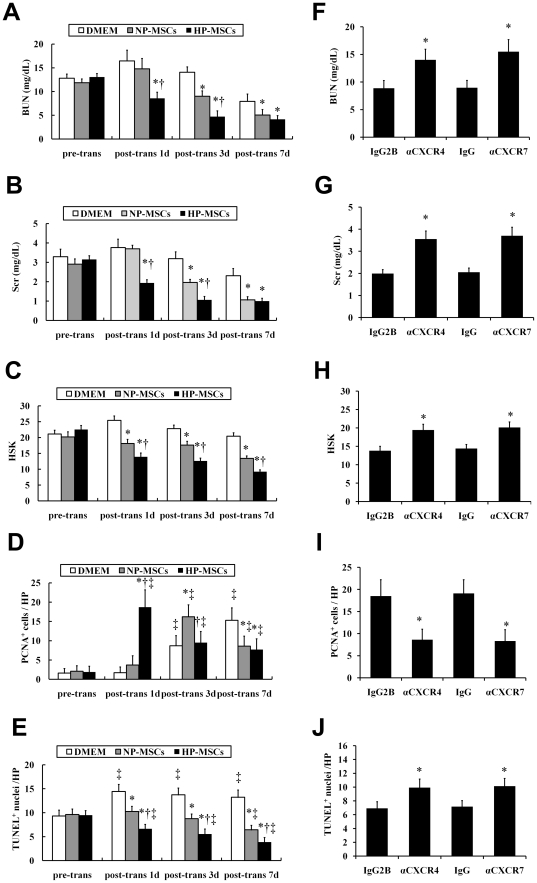
The effects of SDF-1-CXCR4/CXCR7 pathway on the therapeutic efficacy of HP-MSCs for treatment of I/R-AKI. (A and B) BUN (A) and Scr (B) levels as measured in I/R-AKI mice received HP-MSCs, NP-MSCs or vehicle (DMEM). *P<0.05 vs DMEM; ^†^ *P*<0.05 vs NP-MSCs. (C) The histological score of kidney (HSK) in I/R-AKI mice received HP-MSCs, NP-MSCs or vehicle, respectively, was calculated. *P<0.05 vs DMEM; ^†^<0.05 vs NP-MSCs. (D and E) HP-MSC-treated mice showed a significantly earlier rise in proliferating cells (D), and simultaneous reduction of number of apoptotic cells compared with NP-MSC-treated mice (E). *P<0.05 vs DMEM; ^†^ *P*<0.05 vs NP-MSCs; ^‡^ *P*<0.05 vs pre-transplantation. (F through J) BUN levels (F), Scr levels (G), HSK (H), renal PCNA expression (I), and renal TUNEL-apoptosis (J) were evaluated in mice treated with HP-MSCs+IgG2B-isotype control antibody, HP-MSCs+anti-CXCR4 antibody, HP-MSCs+IgG-isotype control antibody, and HP-MSCs+anti-CXCR7 antibody, respectively. *P<0.05, vs the respective isotype-matched control antibodies.

24 h after surgery (i.e. immediately after MSC transplantation), renal function was identically aggravated in animals designated to receive HP-MSCs, NP-MSCs or vehicle treatment, as assessed by blood urea nitrogen (BUN) and serum creatinine (Scr) levels (Fig. 7A and B). Administration of NP-MSCs improved the renal function in animals at days 3 and 7 after transplantation, compared with that in vehicle-treated animals (Fig. 7A and B). However, HP-MSC-treated animals had significantly lower BUN and Scr levels at 24 h after infusion compared with both vehicle- or NP-MSC-treated animals, and the renal function was restored to normal levels at 3 days after transplantation (Fig. 7A and B).

To further substantiate above-mentioned heartening results, the histologic examinations including histological score of kidney (HSK), PCNA and TUNEL staining were evaluated immediately or at days 1, 3, and 7 after transplantation. As expected, compared with control kidneys from vehicle-treated mice, kidneys from either HP-MSC- or NP-MSC-treated mice had significantly reduced HSK, increased number of PCNA-positive cells, and decreased number of apoptotic cells on TUNEL assay (Fig. 7C through 7E). Interestingly, 24 h after cell infusion, the number of PCNA-positive cells in kidneys from HP-MSC-treated mice was significantly increased compared with that from both vehicle-(+11-fold) or NP-MSC-(+5-fold) treated mice (Fig. 7D). The increase in renal cell survival following MSC administration was confirmed by measure of apoptosis using TUNEL analysis. One, three and seven days after intravenous administration of MSCs, the number of apoptotic renal cells detected at the ischemic kidneys was significantly lower in HP-MSC-treated animals than in NP-MSC-treated animals (Fig. 7E).

### Neutralization of either CXCR4 or CXCR7 impairs the improved therapeutic potential of HP-MSCs

To assess whether the SDF-1-CXCR4/CXCR7 axis was involved in the therapeutic potential of HP-MSCs, the cells were injected into the tail vein of I/R-AKI mice after pretreatment with a neutralizing anti-CXCR4 antibody, or an anti-CXCR7 antibody, or their respective isotype-matched antibodies. At day 1 after cell infusion, mice implanted with HP-MSCs pretreated with either anti-CXCR4 or anti-CXCR7 antibody had significantly higher BUN and Scr levels (Fig. 7F and G), HSK (Fig. 7H), as well as number of apoptotic cells in TUNEL assay (Fig. 7J) compared with mice implanted with cells pretreated with theirs respective isotype-matched antibodies. Furthermore, pretreatment of HP-MSCs with either anti-CXCR4 or anti-CXCR7 antibody significantly reduced the number of PCNA-positive cells in kidneys from I/R-AKI mice (Fig. 7I).

## Discussion

**Figure 8:**
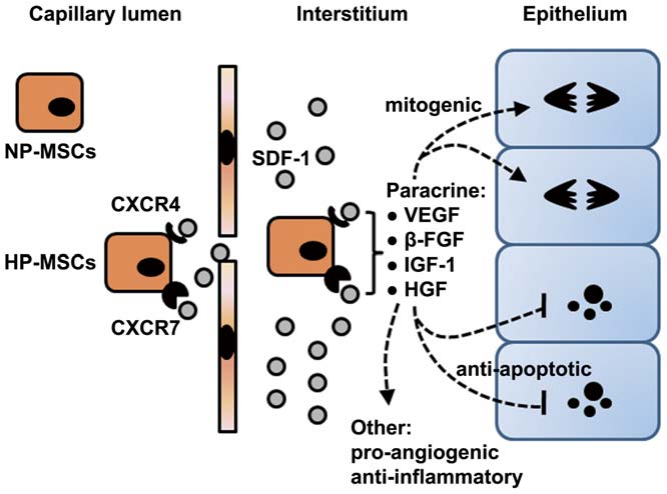
A model of regenerative potential of HP-MSCs in repair of I/R-AKI. Chemokine SDF-1 expression is upregulated in postischemic kidneys. HP enhances the expression of both SDF-1 receptors, CXCR4 and CXCR7, in MSCs. Intravenously injected HP-MSCs are recruited to the ischemic kidney and localized within the injured capillaries and in the interstitium through SDF-1α-CXCR4 interaction. The binding of SDF-1 to both CXCR4 and CXCR7 is responsible for the production of paracrine mediators, including VEGF, β-FGF, IGF-1 and HGF that exert mitogenic, anti-apoptotic, pro-angiogenic, and anti-inflammatory effects.

The present study found for the first time that HP increased the chemotaxis, viability and paracrine actions of MSCs *in vitro* via a mechanism that is dependent, at least in part, on SDF-1-CXCR4/CXCR7 pathway. When administered systemically, the HP-MSCs homed to the ischemic kidney more efficiently than NP-MSCs, which led to significantly improved renal function, accelerated mitogenic response, and reduced cell apoptosis. Importantly, these *in vivo* effects were largely abolished by either CXCR4 or CXCR7 inhibition, indicating that the *in vivo* benefits of HP are also mediated by the SDF-1-CXCR4/CXCR7 axis (Fig. 8).

Although MSCs are able to withstand hypoxia for up to a few days by upregulating survival pathways and increasing glycolytic metabolism [41], they need to survive longer for maintaining a long-term, effective MSC-based therapy in ischemic tissue. Short-term exposure of MSCs to hypoxia can significantly enhance their viability *in vitro* and *in vivo*, and thus improve their tissue repair capabilities after transplantation into the ischemic tissue [38], [42], [43]. HP can enhance the paracrine/autocrine effects of MSCs by altering trophic factor release [38], [43]–[45] and it also plays an critical role in recruiting MSCs to the sites of injury *in vivo* by upregulating the membrane markers associated with migration and homing of MSCs [35]–[37], [46]. A selective *in vivo* expression of chemokine SDF-1 in ischemic tissue in direct proportion to reduced oxygen tensions has been confirmed [46]–[49]. Our data also demonstrated that SDF-1α is upregulated in the ischemic kidneys within hours of I/R and remains elevated for several days. The interaction of locally produced SDF-1 and its receptor CXCR4 expressed on the MSC surface plays an crucial role in the homing of transplanted cells [35], [46], [47], [50], [51]. However, culture-expanded MSCs progressively downregulate CXCR4 expression and lose their ability to migrate toward the SDF-1 gradient in the ischemic tissue [32], [33], [52]. Our previous study reported HP-induced expression of CXCR4 and CXCR7, and the role of both SDF-1 receptors in enhanced migration, adhesion and survival of HP-MSCs *in vitro* [39]. Thus, we used HP as a strategy to enhance the homing of systemically delivered MSCs to the ischemic kidney. Here, we further investigated the effects of SDF-1-CXCR4/CXCR7 axis on the homing and the therapeutic outcome of MSCs *in vivo*.

We first evaluated the role of SDF-1-CXCR4/CXCR7 axis in chemotaxis, viability and paracrine actions of MSCs *in vitro*. To this end, the HP-MSCs that had markedly upregulated expression of CXCR4 and CXCR7 were pretreated with the neutralizing antibody against CXCR4 or CXCR7 to block their functions, and NP-MSCs that did not have detectable CXCR4 and CXCR7 expression were transfected with sense expression vector of CXCR4 or CXCR7 for upregulating these genes. The function blockage of CXCR4 but not CXCR7 in HP-MSCs suppressed the chemotactic response to SDF-1α, and CXCR4 overexpression increased this chemotaxis of NP-MSCs, suggesting that only SDF-1α-CXCR4 axis is responsible for the chemotaxis of MSCs. Otherwise, our study also found that, at least in MSCs, H_2_O_2_-induced cell apoptosis can be reverted by HP, and this HP-induced survival process is mediated by CXCR7, but not by CXCR4. Overexpression of CXCR7 but not of CXCR4 also reverted H_2_O_2_-induced cell apoptosis of NP-MSCs, suggesting that only SDF-1α-CXCR7 axis is responsible for the viability of MSCs *in vitro*. In addition, the enhanced secretion of VEGF, β-FGF, IGF-1 and HGF of MSCs by HP was inhibited by blocking either CXCR4 or CXCR7, and overexpression of either CXCR4 or CXCR7 markedly increased paracrine actions of NP-MSCs, suggesting that not only the SDF-1-CXCR4 interaction but also the binding of SDF-1 to CXCR7 were required for the paracrine actions of MSCs.

Interestingly, the results of this study also clearly demonstrate that, at least in NP-MSCs, SDF-1α preconditioning has no effect on the expression of CXCR4 and CXCR7, *in vitro* chemotaxis, viability and paracrine actions. However, SDF-1α preconditioned HP-MSCs are markedly protected against H_2_O_2_, and this SDF-1α-induced survival process is mediated by CXCR7, but not by CXCR4. Similarly, SDF-1α induced significant increase in the viability of CXCR7-transfected but not of CXCR4-transfected NP-MSCs under H_2_O_2_ culture conditions. Furthermore, SDF-1α also markedly increased the secretion levels of VEGF, β-FGF, IGF-1 and HGF in HP-MSCs and in either CXCR4- or CXCR7-transfected NP-MSCs. These data suggest the role of autocrine SDF-1α under hypoxia and further support the possibility that CXCR4 and CXCR7 play an essential, but differential role in regulating chemotaxis, viability and paracrine actions of MSCs *in vitro*.

In agreement with observations from *in vitro* chemotaxis assay, HP can enhance the homing capacity of MSCs toward the injured kidney in I/R-AKI mice, and this improved capacity is significantly reduced by neutralization of CXCR4, but not of CXCR7. Furthermore, significantly greater numbers of intravenously infused HP-MSCs were homed in the ischemic kidneys than that of NP-MSCs, leading to significantly improved renal function, accelerated mitogenic response, and reduced HSK and apoptotic index. Therefore, that systemic administration of HP-MSCs that have significantly higher expression of CXCR4 and CXCR7 than NP-MSCs may be a useful noninvasive therapy to promote renal repair after I/R. Importantly, these beneficial effects of HP-MSCs on renal tissue regeneration following I/R are largely abolished by a neutralization of either CXCR4 or CXCR7, indicating that SDF-1-CXCR4/CXCR7 axis qualifies as an attractive target for MSC-based therapies. These findings provide new insights into the role of SDF-1-CXCR4/CXCR7 in HP-MSCs for regenerative medicine.

## Materials and Methods

### Ethics Statement

C57BL/6 mice were provided by the Experimental Animal Center of the Fourth Military Medical University (Xi'an, China) and Medical College of Xi'an Jiaotong University (Xi'an, China). This study was carried out in strict accordance with the Guidelines on the Care and Use of Laboratory Animals issued by the Chinese Council on Animal Research and the Guidelines of Animal Care. All procedures involving animals were approved by the Institutional Animal Care and Use Committees of both the Fourth Military Medical University and the Xi'an Jiaotong University. All efforts were made to minimize animals' suffering and to reduce the number of animals used.

### Isolation and culture of MSCs

MSCs were isolated from C57BL/6 mice as previously described [39]. Briefly, femurs and tibiaes were prepared from 4–6-week-old male mice. The marrow was extruded with L-DMEM (Gibco, Grand Island, NY, USA) and cultured in L-DMEM containing 10% fetal bovine serum (FBS) and 1% antibiotic/antimycotic solution (Gibco). After 24 h, the nonadherent cells were removed by replacing the medium. Adherent MSCs had a typical spindle-shaped appearance and were used at passage 3. For characterization of mouse MSCs, cultured cells were subjected to flow cytometry using CD34, CD45, CD90 and CD105 markers (BD Pharmingen, San Diego, CA, USA), and were identified as CD90^+^/CD105^+^ and CD34^−^/CD45^−^cells.

### HP of MSCs

MSCs were cultured in a hypoxia chamber incubator (catalog No. 27310; StemCell Technologies, Vancouver, BC, Canada) at 37°C in 3% O_2_, 5% CO_2_ and 92% N_2_ for 24 h, and these MSCs were named as hypoxia-preconditioned MSCs (HP-MSCs). Normoxia-preconditioned (for 24 h in 95% air, 5% CO_2_) MSCs (named as NP-MSCs) were used as a control.

### Semiquantitative RT-PCR analysis

Total RNA was extracted from bone marrow mononuclear cells (BMMCs) and bone marrow-derived MSCs using Trizol reagents (Invitrogen Life Technologies) according to manufacturer's instructions. The sequence of primers for PCR was as follows: CXCR4, 5′-AAAGCTAGCCGTGATCCTCA-3′ (sense) and 5′-CACCATTTCAGGCTTTGGTT -3′ (anti-sense); CXCR7, 5′-TCACCTACTTCACCGGCACC-3′ (sense) and 5′-ACATGGCTCTAGCGAGCAGG-3′ (anti-sense); SDF-1α, 5′-AAACCAGTCAGCCTGAGCTAC-3′ (sense) and 5′-TTACTTGTTTAAAGCTTTCTC-3′ (anti-sense); GAPDH, 5′-ACCACAGTCCATGCCATCAC-3′ (sense) and 5′-TCCACCACCCTGTTGCTGTA-3′ (anti-sense). The PCR conditions were as follows: denaturation at 94°C for 30 s, annealing at 60–62°C for 30 s, and extension at 72°C for 30 s, which was repeated for 35 cycles. The PCR amplicons were then separated on 2.0% agarose gel by electrophoresis and analyzed by densitometry.

### Western blots

BMMCs and MSCs were washed with ice-cold PBS and scraped in RIPA lysis buffer including protease inhibitors. After loaded and separated on sodium dodecyl sulphate polyacrylamide gel (SDS–PAGE), the proteins were electrophoretically transferred onto a polyvinylidene difluoride membrane and then blocked with 1×TBS plus Tween 20 (TBST) containing 5% nonfat dry milk for 2 h at room temperature. The membrane was incubated overnight at 4°C with SDF-1α (eBioscience, 1∶1000), CXCR4 (Santa-Cruz Biotechnology, 1∶250), CXCR7 (R&D Systems, 1∶200) or β-actin (Abcam, 1∶2000) antibodies appropriately diluted in 1×TBST containing 5% nonfat dry milk. The immune complexes were visualized with appropriate horseradish peroxidase-conjugated secondary antibodies and enhanced chemiluminescence Plus kit (Amersham, Freiburg, Germany).

### ELISA

The production of SDF-1α, VEGF, β-FGF, IGF-1, and HGF in the supernatants of MSCs and in the kidney cortex was determined by ELISA using a commercially available ELISA kit (R&D Systems, Minneapolis, MN, USA) according to the manufacturer's recommendation. Supernatants were prepared by collecting serum-free DMEM medium after 24 hour culture of approximately 1×10^6^ MSCs under normoxia or hypoxia. Decapsulated kidney cortex tissues were retrieved before (n = 10) and 0.5, 1, 3, 5, 7, 14 days after I/R (n = 10) and cell and tissue lysates were obtained by mincing, sonicating, and lysing with RIPA buffer. Protein was quantified by BCA protein assay reagent assay (Pierce, Rockford, IL, USA). Optical density was measured at 450 nm with wavelength correction at 570 nm. All samples and standards were measured in duplicate. In some experiments, MSCs were pretreated with SDF-1α (50 ng/ml), a neutralizing anti-CXCR4 antibody (10 µg/ml, Clone 247506, Rat IgG2B, R&D Systems), rat IgG2B isotype control (10 µg/ml, Clone 141945, R&D Systems), an anti-CXCR7 antibody (10 µg/ml, Catalog number AF4227, sheep IgG, R&D Systems), or a sheep IgG isotype control (10 µg/ml, Catalog number 5-001-A, R&D Systems).

### Chemotaxis assay

The transwell system was purchased from Millipore Inc (Billerica, MA). Briefly, SDF-1α (10 ng/ml, Millipore, Billerica, MA) was placed in the lower chamber, and 10^5^ cells were added to the upper chamber in the presence or absence of a neutralizing anti-CXCR4 antibody, an anti-CXCR7 antibody, a rat IgG2B isotype control antibody, or a sheep IgG isotype control antibody (all at a concentration of 10 µg/ml). The chemotaxis chambers were then incubated for 12 h at 37°C. Then, non-migrating cells were removed from the top chamber, and migrated cells were fixed in methanol and stained with 2% toluidine. The number of cells that had migrated through to the underside of the insert membranes was calculated by counting at least five random separate fields (400-fold magnification).

In some experiments, primary renal tubule epithelial cells (TECs) from 1–2-week-old C56BL/6-mice were cultured in serum-free DMEM and exposed to sublethal hypoxia for 6 h (<3% O_2_) followed by 12 h of reoxygenation (21% O_2_). After hypoxia/reoxygenation (H/R) the cells were washed twice and added in the lower chamber for the transfilter assay. Primary mouse proximal TECs were generated as previously described [53]. Kidney medulla was discarded, kidney cortices was minced and digested with collagenase I (Sigma Chemical Co.). The cell suspensions were filtered through 40 µm strainers (BD Falcon 2350; BD Pharmingen, San Diego, CA, USA) and seeded on Nunclon–treated 6-well plates (Nalgene/Nunc International, Rochester, NY). Once confluent and prior to use, the epithelial nature of the cells was characterized by positive staining for megalin and aquaporin-1 (Santa Cruz Biotechnology, Inc.).

### Cell viability and cytotoxicity assays

Cells in the dish (10^5^ cells/well) were cultured with SDF-1α (50 ng/ml) or SDF-1α plus H_2_O_2_ (250 µM, Sigma-Aldrich) for 6 h in presence or absence of a neutralizing anti-CXCR4 antibody, an anti-CXCR7 antibody, a rat IgG2B isotype control antibody, or a sheep IgG isotype control antibody (all at a concentration of 10 µg/ml). The following standard cytotoxicity tests were performed as described [54]. The viability of the cells was measured using both 3-(4,5-dimethyl-2-thiazolyl)-2,5-diphenyl-2H-tetrazolium bromide (MTT) assay and an automated cell counter (NucleoCounter, New Brunswick Scientific, Edison, NJ). The cell counter technique uses propidium iodide (PI), which binds to cellular nuclei. Depending on sample preparation, the counts provide the total number of cells or viable cells. Cytotoxicty was also determined by measuring the amount of lactate dehydrogenase (LDH) released in the cell culture medium using a Sigma assay kit by measuring absorption at 340 nm.

### Transient cell transfection

The NP-MSCs that had undetectable CXCR4 and CXCR7 expression were transfected using pORF9-mCXCR4 (an expression vector containing the mouse CXCR4 open-reading frame) or pORF9-mCXCR7 (an expression vector containing the mouse CXCR7 open-reading frame), respectively (InvivoGen, CA, USA). Both mCXCR4 and mCXCR7 genes consist of an intronless ORF from the ATG to the stop codon. The ORF size was 1089 bp and the cloning fragment size was 1069 bp for mCXCR4 or 1203 bp for mCXCR7. The protocol for growth of pORF-transformed bacteria and the selection of bacterial clones has been previously described [55]. Briefly, the lyophilized *E.coli* were resuspended with 1 ml of LB medium and streaked onto ampicillin LB agar plate prepared with the *E. coli* Fast-Media Amp agar and incubated at 37°C overnight. Isolated single colony of the bacteria was grown in TB medium supplemented with ampicillin using the Fast-Media Amp liquid overnight at 37°C. The pORF plasmid DNA was extracted using the QUIAGEN plasmid midi kit (Quiagen, USA) and the yield was measured spectrophotometrically. For each transfection, NP-MSCs (2×10^6^) were plated in one well of 6-well plates and grown in DMEM containing 10% FBS until cell density reached 75% confluence. The expression plasmid DNA [15 µg in 800 µl of opti-MEM medium (Gibco, USA)] was mixed with 15 µl lipofectAMINE (Gibco, USA) and incubated at room temperature for 30 min. The MSCs were washed with serum-free medium, mixed with lipofectAMINE/DNA mixture and incubated at 37°C for 5 h and for a further 48 h in the presence of growth medium containing 20% FBS. As a control, NP-MSCs were similarly transfected with an empty vector (pORF9-MCS, Invivogen, CA, USA). In each experiment, the transfection efficiency was assessed using Western blots of transfected and identically treated non-transfected cells. The transfection efficiency ranged from 74–82% and the viability, as assessed by trypan blue dye exclusion, ranged between 90 and 97%. These cells were further used to investigate the functional characteristics of CXCR4- or CXCR7-transfected NP-MSCs in chemotaxis assay, ELISA assay of secreted VEGF, β-FGF, IGF-1 and HGF, cell viability and cytotoxicity assays.

### Induction of I/R-AKI

Models of I/R-AKI were performed in female 6–8-week-old C57BL/6 mice by clamping both renal pedicles for 30 minutes followed by clamp release to allow reperfusion as described earlier [56]. To ensure complete intravenous administration of MSCs, a 27G canula connected to a short polyethylen-catheter was used. At 24 h after surgery, the cells (1×10^6^/0.5 ml L-DMEM) were infused via the cannulated tube that was then flushed with 0.3 ml L-DMEM in order to infuse cells remaining in the tubing. In order to be able to detect the homing of MSCs to the target tissue, MSCs were labeled with ^111^Indium-oxine (GE Healthcare).

The animal were randomly assigned to one of seven experimental groups (n = 10) as follows: group 1, serum-free L-DMEM; group 2, ^111^Indium-labeled NP-MSCs; group 3, ^111^Indium-labeled HP-MSCs; group 4, ^111^indium-labeled HP-MSCs pretreated with IgG2B isotype control antibody; group 5, ^111^Indium-labeled HP-MSCs pretreated with a neutralizing anti-CXCR4 antibody; group 6, ^111^Indium-labeled HP-MSCs pretreated with IgG isotype control antibody; and group 7, ^111^Indium-labeled HP-MSCs pretreated with a neutralizing anti-CXCR7 antibody. Pretreatment of cells with neutralizing antibodies (10 µg/10^6^cells) was performed by incubating for 30 min on ice.

### In vitro labeling and in vivo homing of transplanted MSCs

Radiotracer labeling of MSCs is simpler than fluorescent labeling, and the traced MSCs can be quantified accurately in tissue. Therefore, the radioactive ^111^Indium was used to label MSCs in this study. The cultured MSCs at passage 3 were incubated with radioactive ^111^Indium (100 µCi/10^6^ cells) for 15 min at room temperature. After repeated centrifugation and wash to assure clearance of any unbound radioactivity, the efficiency of ^111^Indium-radiolabeling MSCs was measured as about 80%, resulting in a specific activity of approximately 80 µCi/10^6^ cells. Preliminary experiments showed that the viability and growth of these labeled MSCs were not adversely affected by this labeling procedure (data not shown). Three hours and 1, 2, 3, 5, 7 and 14 days after MSC transplantation, mice were euthanized with an overdose of pentobarbital and the organs, including kidneys, lungs, heart, spleen and liver, were excised and weighed. Biometric data (body weight and organ weight) of the mice allocated to seven different treatment groups revealed no significant difference (*P*>0.05, data not shown). The radioactivity in each organ was measured using a gamma scintillation counter and adjusted by the half-life of ^111^Indium (2.8 days). To facilitate comparisons, specific tissue distribution of the transplanted MSCs was expressed as the percentage of injected dose per gram of tissue (%ID/g) and percentage injected dose per organ (%ID/organ).

### HSK

The excised kidneys were fixed in phosphate-buffered 10% formalin, sectioned, and then stained with hematoxylin and eosin. Evaluation of histological score of kidney (HSK) was performed in a blind manner by a pathologist. HSK was graded on a 4-point scale [57]: 0 = normal histology; 1 = mild damage [less than one-third of nuclear loss (necrosis) per tubular cross section]; 2 = moderate damage [greater than one-third and less than two-thirds of tubular cross section showing nuclear loss (necrosis)]; 3 = severe damage [greater than two-thirds of tubular cross section shows nuclear loss (necrosis)]. The total score per kidney section was calculated by addition of all 10 scores with a maximum possible injury score of 30.

### Immunohistochemical staining

The tissue sections were subject to immunohistochemical staining for SDF-1α and proliferating cell nuclear antigen (PCNA) immediately and 1, 2, 3 and 7 days after cell transplantation. For immunohistochemical staining, the rabbit specific horseradish peroxidase-diaminobenzidine (HRP-DAB) detection immunohistochemical kit (ab64261, Abcam) was used. Briefly, after deparaffination, four micron sections of kidneys were hydrated by decreasing concentrations of ethanol and incubated with a peroxidase-blocking reagent for 30 min. Before immunostaining, sections for SDF-1α staining were treated with 0.1 mol/L sodium citrate buffer (pH 6.0) in a microwave oven for antigen retrieval. Sections were incubated overnight at 4°C with the primary antibodies, a rabbit polyclonal SDF-1α antibody (1∶200, eBioscience, San Diego, CA, USA) and a rabbit polyclonal FL-261 antibody (1∶200, sc-7907, Santa Cruz Biotechnology, Santa Cruz, USA). Control experiments included omission of either the primary or secondary antibody. The reaction sections were incubated with biotinylated goat anti rabbit IgG(H+L) as a secondary antibody for 10 min. Visualization of the specific binding on the sites of primary antibodies was developed by an enzymatic conversion of the DAB into a brown precipitate by streptavidin peroxidase. After counterstaining with hematoxylin, the sections were mounted, cleared, and coverslipped. The number of PCNA-positive cells, a marker of mitogenesis, was carried out by counting the number of positive nuclei in 10 randomly selected sections of kidney cortex and outer medulla, and converted to the mean number of positive cells in high-power fields (HPF, ×20 magnification). Terminal deoxynucleotidyl transferase dUTP nick end labeling (TUNEL) assay was performed according to the manufacturer's instructions (In Situ Cell Death Detection Kit; Roche China, Ltd.) to detect tubular cell apoptosis in post-ischemic kidney. TUNEL-stained sections were screened for positive nuclei under a fluorescence microscope, and 10 random fields in the corticomedullary area were counted for every kidney at ×40 magnification.

### Statistical Analysis

Each experiment was repeated at least ten times. All values are given as mean ±SD. Student's t test and one-way ANOVA of variance followed by Dunnett's multiple comparison tests were adopted for all statistical data. All analyses were carried out using SPSS 11.0 software (Chicago, IL, USA). *P*<0.05 was considered statistically significant.
